# Short-Term Effects of Chlorpromazine on Oxidative Stress in Erythrocyte Functionality: Activation of Metabolism and Membrane Perturbation

**DOI:** 10.1155/2016/2394130

**Published:** 2016-08-08

**Authors:** Silvana Ficarra, Annamaria Russo, Davide Barreca, Elena Giunta, Antonio Galtieri, Ester Tellone

**Affiliations:** ^1^Department of Chemical, Biological, Pharmaceutical and Environmental Sciences, University of Messina, V. le Ferdinando Stagno d'Alcontres 31, 98166 Messina, Italy; ^2^Virology and Microbiology AOOR Papardo-Piemonte, V. le Ferdinando Stagno d'Alcontres, 98166 Messina, Italy

## Abstract

The purpose of this paper is to focus on the short-term effects of chlorpromazine on erythrocytes because it is reported that the drug, unstable in plasma but more stable in erythrocytes, interacts with erythrocyte membranes, membrane lipids, and hemoglobin. There is a rich literature about the side and therapeutic effects or complications due to chlorpromazine, but most of these studies explore the influence of long-term treatment. We think that evaluating the short-term effects of the drug may help to clarify the sequence of chlorpromazine molecular targets from which some long-term effects derive. Our results indicate that although the drug is primarily intercalated in the innermost side of the membrane, it does not influence band 3 anionic flux, lipid peroxidation, and protein carbonylation processes. On the other hand, it destabilizes and increases the autooxidation of haemoglobin, induces activation of caspase 3, and, markedly, influences the ATP and reduced glutathione levels, with subsequent exposure of phosphatidylserine at the erythrocyte surface. Overall our observations on the early stage of chlorpromazine influence on erythrocytes may contribute to better understanding of new and interesting characteristics of this compound improving knowledge of erythrocyte metabolism.

## 1. Introduction

Chlorpromazine (CPZ) is a neuroleptic drug belonging to the group of phenothiazines widely used in the treatment of some psychiatric disorders [1]. In other nonpsychiatric indications, the drug is used in the symptomatic treatment of vomiting and intractable hiccups and in the treatment of severe pain and is associated with antihistamines during preanesthetic clinical interventions. The tricyclic ring structure of CPZ is hydrophobic, partitioning with relative ease into the bulk hydrocarbon phase of membrane bilayer systems, while the tertiary propylamine tail region of the drug is hydrophilic, interacting well with the polar headgroups of membrane bilayers [2, 3]. CPZ penetrates into the acyl region of phospholipid membranes, affecting the acyl chain order [4] and lipid phase transition [5]. Since the inner leaflet of the red blood cells plasma membrane contains mostly anionic phospholipids [6, 7], electrostatic attraction forces make CPZ mainly localized in this site. In detail, CPZ has been found to interact preferentially with bilayers containing phospholipids with a high portion of phosphatidylserine (PS) and highly unsaturated acyl chains and the binding to the PS in the bilayer enhances phospholipid headgroup mobility. Because CPZ accumulates at the inner leaflet of the membrane bilayer, it may also modulate the activity of membrane-associated enzymes that are involved in phosphoinositide turnover. Moreover, CPZ, through direct molecular interaction, inhibits human erythrocyte acetylcholinesterase (AchE), generally used as a marker of membrane integrity; this phenomenon may be related to formation of CPZ micellar aggregates [8, 9].

In the human body, the blood is one of the most important and abundant connective tissues involved in the CO_2_ elimination and in the body defense. Besides, it is generally recognized that red blood cells (RBCs) not only are well-designed vehicles for O_2_ transport to the metabolic tissues but also function as oxygen-sensing regulating O_2_ distribution in the microcirculation.

For all these reasons and because of their abundance and easy availability, RBCs were used as an experimental model to elucidate the mode of action of CPZ particularly with respect to its interaction with the erythrocyte membrane and the possible interference with normal metabolic events that occur inside the cell. In this context, particular emphasis will be given to the potential CPZ modulation of band 3 protein (PB3) also in the light of its preferential binding to the T-state of human adult hemoglobin (Hb) [10]. In fact, it should be recalled that PB3 regulates anion exchange and erythrocyte metabolism by interacting competitively through its N-terminal end with some glycolytic enzymes (GEs) and Hb. This allows an adequate and timely glucose-6-phosphate (G6P) flux towards the pentose phosphate pathway (PPP) in the high-oxygenation state (HOS) or to the glycolytic one (EMP) in the low-oxygenation state (LOS) [11–13]. The correct modulation of the G6P metabolic fluxes (centred on the T-R states of Hb) protects RBCs against oxidative stress and maintains the structural and functional integrity of the cells, since the increase of the oxidative level in RBCs promotes the caspase 3 activation, which is followed by the cleavage of band 3 protein cytoplasmic domain (cdb3) with loss of metabolic regulation and with almost the suppression of the release of ATP [14–18].

There exists a rich literature concerning the side effects, therapeutic effects, and/or complications due to CPZ; however most of these studies investigate the influences of long-term treatment with the drug. The purpose of this paper is to focus on the short-term effects of CPZ on RBCs; then we tested the influence of the drug on the membrane (PB3 anion exchange, ATP release, lipid peroxidation, PS exposure, and hemolysis degree), cytoplasmic proteins (hemoglobin, caspase 3), and antioxidant or prooxidant state of RBC (proteins carbonylation processes, oxidation of glutathione, and superoxide anion generation). This paper will help to clarify the specific biochemical interaction through which the drug produces its short-term pharmacological effect and may help to open new potential therapeutic targets.

## 2. Materials and Methods

### 2.1. Materials

All reagents were purchased from Sigma-Aldrich (St. Louis, MO, USA). Citrate fresh human blood was obtained from informed healthy donors who declared that they had abstained from all drug treatments for at least one week prior to sample collection, in accordance with the principles outlined in the Declaration of Helsinki. Concentrated stock solution of CPZ was prepared by dissolving the drug in ethanol. On the basis of the CPZ concentrations observed in vivo at therapeutic doses, the drug concentrations range chosen for selected experiments was 10–100 *μ*M.

### 2.2. Preparation of Erythrocytes

Citrate blood samples were washed three times with an isoosmotic NaCl solution. During washing, white blood cells were discarded from the pellet. After washing, the RBCs were resuspended (haematocrit 3%) in the incubation buffer (35 mM Na_2_SO_4_, 90 mM NaCl, 25 mM HEPES [N-(2-hydroxyethyl)-piperazine-N1-2-ethanesulfonic acid], and 1.5 mM MgCl_2_), adjusted to pH 7.4 or 7.3 and 310 ± 20 mOsmol per kg, measured by an Osmostat OM-6020 apparatus (Daiichikagakuco, Kyoto, Japan). In experiments performed with deoxygenated erythrocytes, different levels of deoxygenation (from 15% up to 90%) were checked by determining Hb saturation spectrophotometrically (Beckman DU 640 spectrophotometer) using the millimolar absorptivities reported by Zijlstra et al. [22]. The buffer used to prepare deoxygenated erythrocytes was 0.1 pH unit lower than that used for oxygenated erythrocytes, in order to compensate for the Haldane effect that occurs during deoxygenation [23]. Methemoglobin (met-Hb) levels and the degree of hemolysis were determined at the end of the incubation time as reported by Zijlstra et al. [22].

### 2.3. Kinetic Measurements

Cells were incubated in the above incubation buffer at 25°C, under different experimental conditions in the presence and absence of CPZ at concentrations ranging from 50 up to 150 *μ*M. At several time intervals (5, 15, 30, 60, 90, and 120 min), 10 *μ*mol of the stopping medium SITS (4-acetamido-40-isothiocyanostilbene-2,20-disulfonic acid) was added to each test tube containing the RBC suspension at periodic time intervals. Cells were separated from the incubation medium by centrifugation (J2-HS Centrifuge, Beckman, Palo Alto, CA, USA) and washed three times at 4°C with a sulphate-free medium to remove the sulphate trapped on the outside. After the final washing, the packed cells were lysed with perchloric acid (4%) and distilled water. Lysates were centrifuged for 10 min at 4000 ×g (4°C) and membranes were separated from the supernatant. Sulphate ions were precipitated from the supernatant by adding a glycerol/distilled water mixture (1 : 1, V/V), 4 M NaCl and 1 M HCl, and 1.23 M BaCl_2_·2H_2_O to obtain a homogeneous barium sulphate precipitate. The absorbance of this suspension was measured at 350–425 nm. Sulphate concentration was determined using a calibrated standard curve, obtained by measuring the absorbance of suspensions with known amounts of sulphate [24]. The experimental data on sulphate concentration as a function of the incubation time were analyzed using the following equation: *c*(*t*) = *c*_*∞*_(1 − *e* ^−*kt*^), where *c*(*t*) represents sulphate concentration at time *t*, *c*_*∞*_ is intracellular sulphate concentration at equilibrium, and *k* is the rate constant of sulphate influx.

### 2.4. Intracellular CPZ Determination

Washed RBCs were incubated at 37°C for 2 h with 100 *μ*M CPZ in the above incubation buffer. Samples were washed and the packed cells were lysed with 10% ethanol. Lysates were centrifuged for 10 min at 4000 ×g (4°C) and the supernatant was filtered with 0.45 mm PTFE filter. Free CPZ was analyzed by High Performance Liquid Chromatography (HPLC) with a Shimadzu system, consisting of an LC-10AD pump system and an SPDM10A diode array detector, a Rheodyne 7725i injector with a 20 mL sample loop, and a reverse-phase Supelco C18 column (5 mm, 250 × 4.6 mm). The mobile phase was acetonitrile : water (70 : 40 v/v). The flow rate was 1.0 mL/min at 25°C. CPZ was detected at 286 nm and determined by comparison of peak areas with a standard solution of 100 *μ*M CPZ.

### 2.5. Measurement of Intra-Extracellular ATP

ATP was measured by the luciferin-luciferase technique [19, 25] in which the amount of light generated by the reaction of ATP with firefly tail extract is dependent on the ATP concentration. Sensitivity was augmented by the addition of synthetic D-luciferin to the crude firefly tail extract. A 200 mL sample of the RBC suspension was injected into a cuvette containing 100 mL of crude firefly tail extract (10 mg/mL distilled water, FLE 250; Sigma-Aldrich) and 100 mL of a solution of synthetic D-luciferin (50 mg/100 mL distilled water; Sigma-Aldrich). The light emitted was detected using a luminometer (1251 Bio Orbit luminometer). A standard curve was obtained on the day of each experiment. For ATP released from cells, after CPZ 50 *μ*M incubation, erythrocytes were diluted 1 : 100 and incubated with the direct activator of Gi, Mastoparan 7 (4 *μ*M Mas 7). To measure the total intracellular ATP of erythrocytes all the ATP consuming processes were blocked deproteinizing samples with trichloroacetic acid (TCA). ATP was measured as described above, after dilution of TCA to 0.01% in order to avoid interference with the assay. Values were normalized to ATP concentration per erythrocyte.

To exclude the presence of significant hemolysis in studies where the release of ATP was measured, samples were centrifuged at 3000 ×g at 4°C for 5 min and the presence of free Hb in the supernatant was determined by light absorption at a wavelength of 576 nm.

### 2.6. Measurement of Hemolysis

The hemolysis of erythrocytes was determined spectrophotometrically at 576 nm based on the ratio of Hb released from cells to the total cellular Hb content after hemolysis with distilled water. The ratio of hemolysis was calculated from the equation

(1) $$\begin{array}{c} H\left(\;\%\;\right)=\frac{A_{1}}{A_{2}}*100\%, \end{array}$$

where *H*(%) is the percent of hemolysis of the erythrocytes, *A*_1_ is the absorbance of the supernatants of the samples of the erythrocytes incubated with or without CPZ, and *A*_2_ is the absorbance of the supernatant of the samples after complete hemolysis with distilled water.

### 2.7. Methemoglobin (Met-Hb) Determination

Washed RBCs were treated with CPZ (25, 50, and 100 *μ*M), at different incubation times from 15 to 90 min, lysed with distilled water and freezing at −20°C, and then centrifuged at 18,000 rpm for 30 min. The percentage of met-Hb was determined spectrophotometrically in a range of wavelengths from 500 to 680 nm [22].

### 2.8. CPZ Effects on Superoxide Anion Generation

Superoxide anions were generated to the references according to methods present in the literature with minor modifications [26, 27]. The reaction mix was made up of 1 mL nitroblue tetrazolium (NBT) solution (156 mM NBT in 100 mM phosphate buffer, pH 7.4), 1 mL NADH solution (468 mM in 100 mM phosphate buffer, pH 7.4), and increasing concentrations (0–100 *μ*M) of CPZ. The reaction was started by adding 100 *μ*L of phenazine methosulphate (PMS) solution (60 mM PMS in 100 mM phosphate buffer, pH 7.4) to the mixture. The reaction mixture was incubated at 25°C for 5 min, and absorbance at 560 nm was measured against a blank sample. Decreased absorbance of the reaction mixture indicated increased superoxide anion scavenging activity.

### 2.9. Caspase 3 Assay

Citrate blood samples were washed three times with an isoosmotic NaCl solution. The white blood cells were discarded from the pellet during washing [28]. After washing, the RBCs were resuspended (haematocrit 3%) in the incubation buffer (35 mM Na_2_SO_4_, 90 mM NaCl, 25 mM HEPES, and 1.5 mM MgCl_2_), adjusted to pH 7.4, and incubated for 2 h at 37°C in the absence or in the presence of 100 *μ*M of CPZ or 100 *μ*M of* tert*-butyl-hydroperoxide (t-BHT). After treatment, erythrocytes were collected by centrifugation at 3000 rpm for 5 min, resuspended in HEPES buffer [100 mM HEPES pH 7.5, 20% glycerol, 5 mM DTT, and 0.5 mM ethylenediaminetetraacetic acid (EDTA)], and lysed by sonication. The cell lysates were clarified by centrifugation at 15,000 rpm for 10 min. The supernatant was passed through Microcon YM 30 (Nominal Molecular Weight Limit 30,000) to obtain a partial purification of caspase 3. The cell lysates (100 mL) were incubated at 37°C for 1 h with enzyme-specific colorimetric substrates (Ac-DEVD-pNA 100 mM in HEPES buffer) in a final volume of 600 mL [29]. Caspase 3 activity was analyzed with a spectrophotometer after pNA release at 405 nm and is expressed as the n-fold value of the untreated sample.

### 2.10. Lipid Peroxidation Assay

Erythrocytes isolated as described in Barreca et al. [30] were incubated for 2 hour in the absence or in the presence of 100 *μ*M CPZ and 100 *μ*M of t-BHT or in the presence of both compounds. After incubation, the samples were washed three times with 10 volumes of 0.9% NaCl and centrifuged at 2500 rpm for 5 min. During the last wash, the packed cells were resuspended in 30 volumes with ice-cold hypotonic medium containing 5 mM Tris and 5 mM KCl to yield hemolysate and then centrifuged for 10 min at 12,000 rpm. This operation was repeated three times. Last, the hemolysate was resuspended in 0.9% of NaCl and used for lipid peroxidation assay by the thiobarbituric acid reactive substances (TBARS) methods [31].

### 2.11. Protein Carbonyl Groups Detection

Carbonyl content is determined by a commercial available Protein Carbonyl Content Assay Kit (Sigma-Aldrich). The protein carbonyl groups have been detected following the derivatization with 2,4-dinitrophenylhydrazine (DNPH). The stable dinitrophenyl (DNP) hydrazone adducts obtained can be detected at 375 nm.

### 2.12. Total Glutathione Analysis

The total glutathione (GSSG + GSH) amounts inside the erythrocyte have been analyzed by a commercial kit (Glutathione Assay Kit, Sigma-Aldrich) following the instruction supplied by the seller.

### 2.13. Detection of Reduced Glutathione

The erythrocytes have been treated as described in the above section. After the red blood cells have been centrifuged for 5 minutes at 1400 ×g and washed twice with 3 volumes of PBS, an aliquot of the red blood cell pellet has been withdrawn and added to the same volume of 5% 5-sulfosalicylic acid solution. The samples have been vortexed vigorously, left for 10 minutes at 2–8°C, and centrifuged at 10,000 ×g for 10 minutes. An aliquot of the supernatant (100 mL) has been added to a reaction mix with a final volume of 1.0 mL composed of potassium phosphate buffer (100 mM pH 7.0), 1.0 mM EDTA, and 80 *μ*M DTNB. The change in absorbance has been monitored at 412 nm and quantified according to a standard curve obtained with known amount of standard glutathione. The results have been presented as fold of control red blood cells incubated in the same experimental condition but without the treatment with CPZ.

### 2.14. Flow Cytometry

RBC suspensions after incubation for 2 h with or without CPZ (50 *μ*M) were diluted to approximately 106 cells/mL and analyzed using a Becton-Dickinson, FACS CANTO II flow cytometer, with simultaneous separate detection at low angle (FSC) and right angle (SSC). The light scattered near the forward direction (low angle) is expected to be proportional to the size (volume) of the particle and is independent of the cell refractive index and shape, whereas scattering at the right angle depends on the cell shape and internal properties of the scattering particles [32]. FSC/SSC is a dual-parameter contour plot histogram proportional to total cell diversity. FSC-A histograms represent the light scattered near the forward direction (proportional to the volume of the particles). Each measurement was done for 30,000 cells. Data were analyzed with DIVA Becton-Dickinson. Expression of CD59 was measured incubating RBCs (0.1% suspension in HEPES buffer) with 20 *μ*L of anti-CD59 resuspended in FACS buffer.

Fluorescence-activated cell sorting (FACS) analysis was performed as described by [33]. RBCs were incubated for 2 h in the presence or absence of CPZ (50 *μ*M) in annexin-binding buffer containing 0.14 M NaCl, 0.01 M HEPES-NaOH (pH 7.4), and 2.5 mM CaCl_2_. Erythrocytes were suspended in a solution composed of Annexin-V-Fluos and annexin buffer. After 10 min of incubation in the dark, samples were finally diluted to 1 : 5 in annexin-binding buffer and measured using flow cytometric analysis. Cells were analyzed by forward scatter, and annexin fluorescence intensity was measured in fluorescence channel FL-1 with an excitation wavelength of 488 nm and an emission wavelength of 530 nm.

### 2.15. Statistical Analysis

Data are presented as mean ± standard deviation (SD). The data were analyzed by one-way analysis of variance. The significance of the differences in relation to the respective controls for each experimental test condition was calculated by Student's *t*-test for each paired experiment. A *P* value of <0.05 was regarded as indicating a significant difference.

## 3. Results

**Figure 1:**
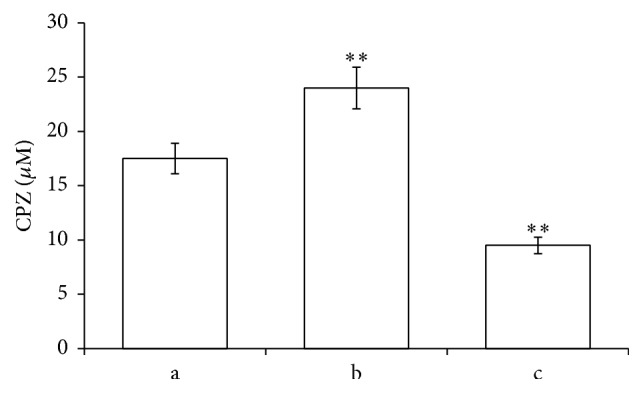
HPLC determination: CPZ distribution outside (a), in the membrane (b), or inside the RBCs (c). Conditions: RBCs were incubated with 100 *μ*M CPZ. This graph derives from the data of two different experiments; for further elucidations see Materials and Methods. ^*∗∗*^Significant differences at *P* < 0.05 versus control.

In order to investigate the influence of CPZ on RBCs, cells incubated with 100 *μ*M CPZ for 2 h at 37°C were analyzed by reverse-phase HPLC separation, revealing that the drug is partly distributed in the cytoplasm but mostly is deposited in the membrane. In detail, a significant amount of CPZ with a percentage comprised between ~20 and ~30% has been found in the RBCs cytoplasm and outside of the membrane, respectively, while almost the double amount (~50%) was intercalated in the membrane (Figure 1). This intercalation of the amphiphilic drug may differently affect the bilayer inducing membrane deformation [34]. Since it is known that mechanical deformations stimulate the release of ATP from RBCs [35], we evaluated the influence of CPZ on ATP release from RBCs comparing the effect to Mas 7 a direct activator of the heterotrimeric G protein. The results shown in Table 1 clearly indicate that RBCs pretreated with CPZ 50 *μ*M released significantly more ATP than in normal conditions and this treatment affects even the intracellular [ATP] that decreases in respect to the control.

Another fundamental element for RBC functionality is PB3. It is one of the most representative proteins of the erythrocyte membrane; we studied the impact of CPZ on the anion transporter PB3.

**Figure 2:**
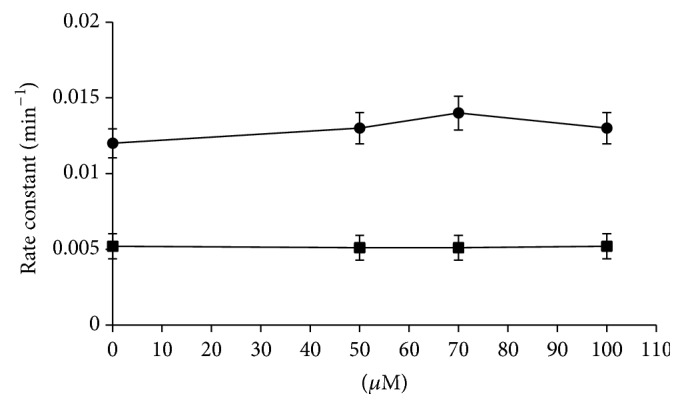
Effect of CPZ (0–100 *μ*M) on the rates of sulphate transport in oxygenated (circles) and deoxygenated (squares) RBCs. Results are from four independent experiments ± SD.

Kinetic measurements of anion flux in RBCs treated with different concentrations of CPZ in the HOS (~90% saturation) and LOS (~15% saturation) revealed that the addition of the drug does not lead to an appreciable increase in the anion exchange even at high concentrations of CPZ (Figure 2).

Moreover, despite the well-known functional interaction between PB3 and Hb [10], the binding of the drug to Hb does not seem to alter in any way the normal function of the anion exchange. However, our experiments evidenced that the Hb-CPZ interaction destabilizes the protein by increasing the autooxidation; in fact as shown in Table 2, RBCs incubated with CPZ showed a met-Hb percentage higher than normal. In detail, met-Hb values were over 20% after 15 min of incubation time and this percentage did not significantly change increasing the CPZ concentration. However, after 90 min of incubation time, we found significant differences (*P* < 0.05) between samples incubated with 25 *μ*M CPZ (over 50% of met-Hb) and those with 50 and 100 *μ*M CPZ (over 60% of met-Hb).

The oxidation of Hb to met-Hb generates significant amount of superoxide that if not immediately neutralized can lead the RBC to an uncontrolled increase in oxidative stress (Fenton and Haber-Weiss reactions) which in turn may activate caspase 3 with a consequent membrane derangement and hemolysis [17].

**Figure 3:**
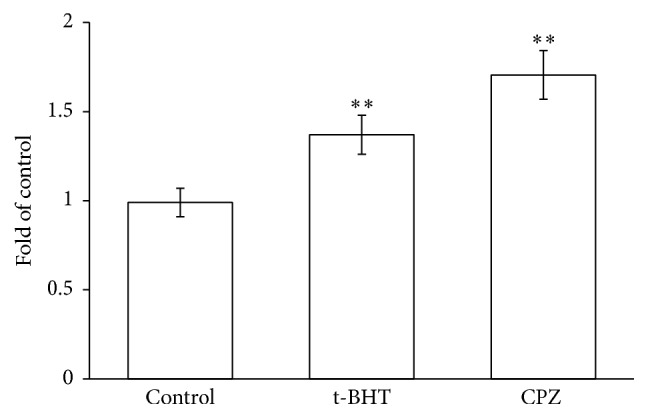
Caspase 3 activity in RBCs incubated in the absence (a control) or in the presence of CPZ 50 *μ*M or t-BHT 100 *μ*M. Results are from four independent experiments ± standard deviation. Asterisks indicate significant differences at *P* < 0.05 versus control.

Caspase 3 activity was measured by incubating RBCs in the absence and in the presence of CPZ 50 *μ*M or t-BHT 100 *μ*M, a well-known oxidant. The experiments shown in Figure 3 clearly indicate that CPZ induces a remarkable activation of caspase 3, with an increase of approximately 1.7-fold with respect to the control, and even superior to t-BHT.

**Figure 4:**
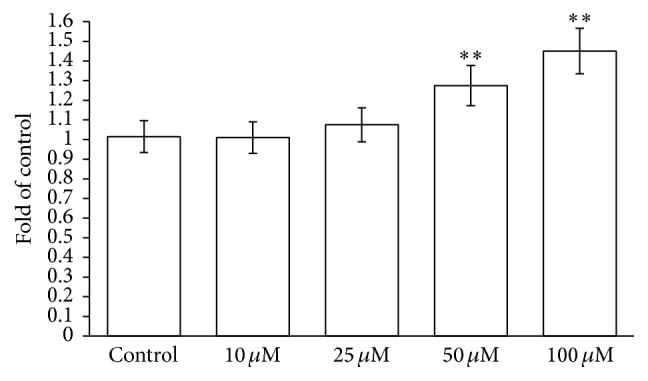
Effects of CPZ (0–100 *μ*M) on superoxide anion radical generation. Each point is the mean value, obtained from three different experiments (*n* = 3). Asterisks indicate significant differences at *P* < 0.05 versus control.

To confirm the increase of oxidative stress induced in RBCs by the drug, the effects of CPZ (0–100 *μ*M) on radicals generation were analyzed.  Figure 4 shows a negligible activity of CPZ in a range of 0–25 *μ*M, but higher concentrations (over 50 *μ*M) caused an increased generation of superoxide anion with respect to the control (of ~1.28- and ~1.44-fold for CPZ 50 *μ*M and 100 *μ*M, resp.).

**Figure 5:**
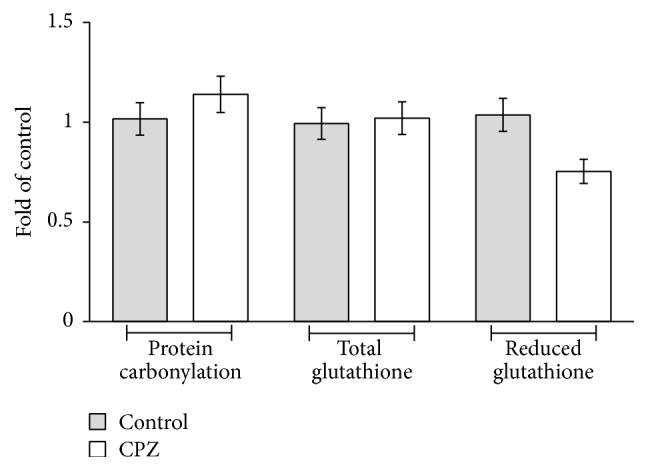
Intracellular levels of protein carbonylation, total glutathione, and reduced glutathione, in RBCs incubated in the absence or in the presence of CPZ 50 *μ*M. Each point is the mean value, obtained from three different experiments (*n* = 3).

These results evidence a prooxidant activity of CPZ inside the erythrocytes and, in our experimental data, it has been further analyzed by detection of carbonyl protein groups, total glutathione, and reduced glutathione amount (Figure 5). Protein carbonylation is commonly studied in those systems where there is an increase in oxidative stress and is due mainly to covalent adduction of lipid aldehydes, produced from hydroperoxidation of polyunsaturated fatty acyl groups, to the side chains of lysine, histidine, and cysteine residues. In erythrocytes treated with CPZ there is a little increase of protein carbonylation corresponding to ~1.1-fold the level found in control erythrocytes that may be speculatively correlated with early stages of lipid peroxidation. Moreover treatment of RBCs for 2 hours in the presence of 100 *μ*M of CPZ did not induce depletion of total glutathione amounts, while there is a net decrease of reduced glutathione level corresponding to ~1.4-fold the level found in control RBCs. In particular the GSH/GSSH ratio has been found to be of ~10.7 in control erythrocytes and it decreased ~5.5-fold in erythrocytes treated with CPZ 50 *μ*M.

**Figure 6:**
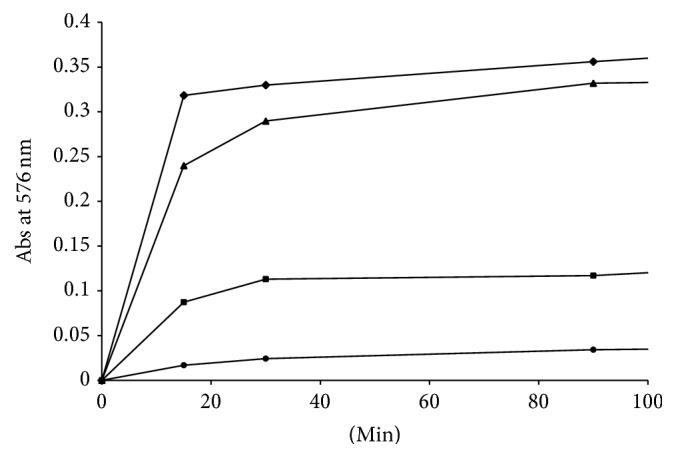
Abs at 576 nm of Hb released from RBCs calculated at different times of incubation of the erythrocytes in the absence (circle symbols) and in the presence of CPZ 25 *μ*M (squares), 50 *μ*M (triangles), and 100 *μ*M (rhombus) *μ*M. Each point is the mean value, obtained from three different experiments (*n* = 3).

**Figure 7:**
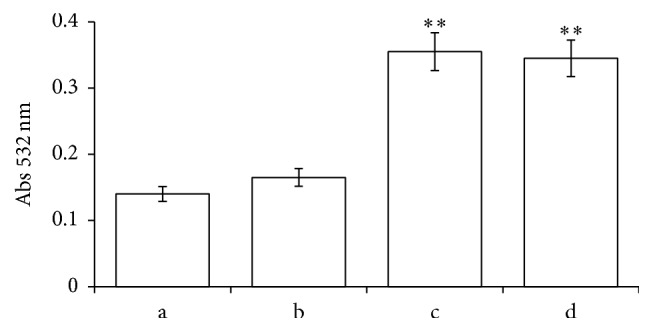
Lipid peroxidation assay of RBC membrane. RBCs were incubated for 2 h in absence (a) or in the presence of CPZ 100 *μ*M (b) or t-BHT 100 *μ*M (c) or both additives CPZ 100 *μ*M and t-BHT 100 *μ*M (d). Each point is the mean value, obtained from three different experiments (*n* = 3). Asterisks indicate significant differences at *P* < 0.05 versus control.

The RBC membrane integrity was further analyzed measuring the percentage of hemolysis, lipid peroxidation, and flow cytometry.  Figure 6 shows RBCs hemolysis induced by increasing drug concentrations after 2 h of incubation time; slight percentage hemolysis was already observed with CPZ 25 *μ*M. On the contrary, no significant levels of membrane peroxidation were measured in the presence of the drug at the maximum concentration utilized in our experiments (Figure 7).

**Figure 8:**
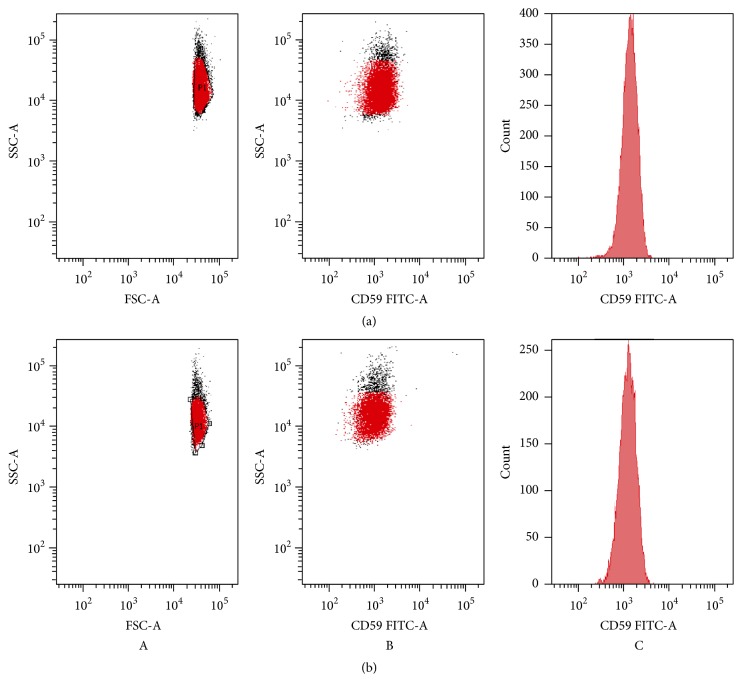
Flow cytometry analysis of CPZ-induced changes in RBC morphology. Scattering diagrams of controls (a) and RBCs incubated for 2 h (b) with CPZ 50 *μ*M (A), with CPZ 50 *μ*M and anti-CD59 (B; C).

The structural integrity of RBC membrane was further analyzed by the expression of the CD59, one of the major complement protective proteins in RBC membranes, anchored by glycosylphosphatidylinositol on the cell surface. CD59-positive cells were detected in all samples after 2 h of incubation time with CPZ 50 *μ*M (Figure 8), and there was no statistically significant difference in the proportion of the CD59 between samples and the control RBCs.

A more specific evaluation of the membrane integrity was performed quantifying the PS exposure at the RBCs surface (based on the fluorescence of RBCs with annexin-V-FITC).

**Figure 9:**
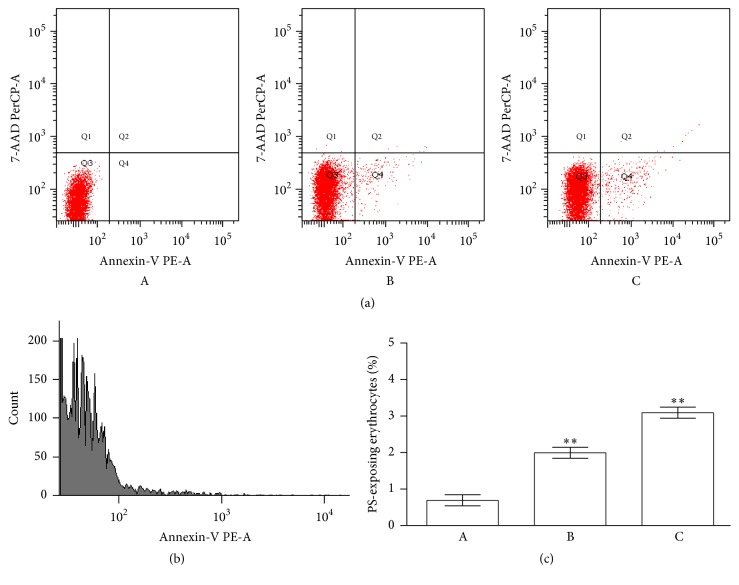
Phosphatidylserine exposure following treatment of RBCs for 2 h with CPZ 50 *μ*M and t-BHT 10 *μ*M. (a) Flow cytometry analysis of annexin-V-binding of erythrocytes following exposure to buffer solution without (A) and with CPZ (B) or t-BHT (C); (b) FACS histogram showing annexin binding in a representative experiment of RBCs incubated with CPZ; and (c) arithmetic means ± SD of erythrocyte annexin-V-binding (*n* = 12) following incubation to buffer solution without (A) or with (B) presence of CPZ; as a control, the effect of BHT is shown (C). ^*∗∗*^Significant differences at *P* < 0.05 versus control.

As shown in Figure 9, treatment of the RBCs with CPZ (50 *μ*M) increased the percentage of annexin-binding cells, detectable in Q4 quadrant, supporting the influences of this drug just in the early phases of the treatment.

## 4. Discussion

Our results show that the CPZ is able to cross the erythrocyte membrane, but probably because of its peculiar chemical structure, it intercalates abundantly in the phospholipid bilayer. Despite this, curiously, unlike what we reported for other molecules [36], CPZ does not cause any functional change in the anion transport. These results are in full agreement with literature data [37]. In particular, Kubota et al. [37] have shown that micromolar concentration of chlorpromazine has no influence on nitrate (NO_3_^−^) movement in isolated RBC or in the whole blood. But it appears quite surprising if it is evaluated that PB3 is highly expressed in the RBC membrane just where the drug is mostly concentrated. The action of CPZ on the membrane has been widely demonstrated by the observation of the induction of stomatocytic transformation, always linked to a decrease in cytoplasmic pH (pHi) [20, 38].

The anion transport stability observed, although essential for disposal of carbonate anion and peroxynitrite, will not be sufficient to guarantee the ionic stability required for the normal RBC life cycle.

The decrease of intracellular ATP strongly penalizes all the ATP dependent pumps such as Na^+^/K^+^ ATPase, H^+^ ATPase, and Na^+^/H^+^ antiport, that is essential for ionic stability, while its release favors the microcirculation through the paracrine action of the nucleotide on purinergic receptors of vascular epithelium and the concomitant synthesis of nitric oxide by nitric oxide synthase.

This may strongly support the low pHi, observed in RBCs tending to a stomatocytic transformation, and the caspase 3 activation always linked to an acidic pHi [20, 38], both conditions observed in the presence of CPZ. This caspase 3 is a zymogen maintained in an inactive structural conformation, by an Asp-Asp-Asp regulatory tripeptide named “safety-catch” [21]. This site is influenced by pH variation and, in particular, it is disrupted by acidification, resulting in an enhanced autocatalytic maturation of the protein. Caspase 3 activation has a critical role in inducing cellular apoptosis and thus it may be utilized for its antiproliferative effects in several cancers therapies [21].

Moreover, the energy depletion and the resulting shift of the metabolic flux of G6P towards the EMP would deprive the PPP of the carbonaceous source indispensable to the production of the reducing power (NADPH) necessary to counteract oxidative stress that RBCs have to face due to the high concentration of prooxidant substrates, such as O_2_ and iron. The resulting change in the redox state of the RBCs towards the oxidation could also further contribute to the activation of caspase 3 [15, 39].

Caspase 3 action, once primed, mediates cleavage of the cdb3 and the Na^+^/H^+^ antiporter, supporting two vicious circles, one that induces oxidative stress (for loss of cdb3, control site for the metabolic fluxes of G6P) and the other one that further increases the intracellular acidity (for loss of activity of the Na^+^/H^+^ antiporter).

What was described could be further exacerbated by a condition similar to the energy depletion caused by calcium entry (scramblase activation) and caspase 3 activation. This leads to a further derangement of the RBC membrane and eryptosis. Furthermore, increase of intracellular [Ca^2+^] causes the detachment of phosphotyrosine phosphatase from PB3 and its inhibition promoting tyrosine phosphorylation of cdb3 [40].

**Figure 10:**
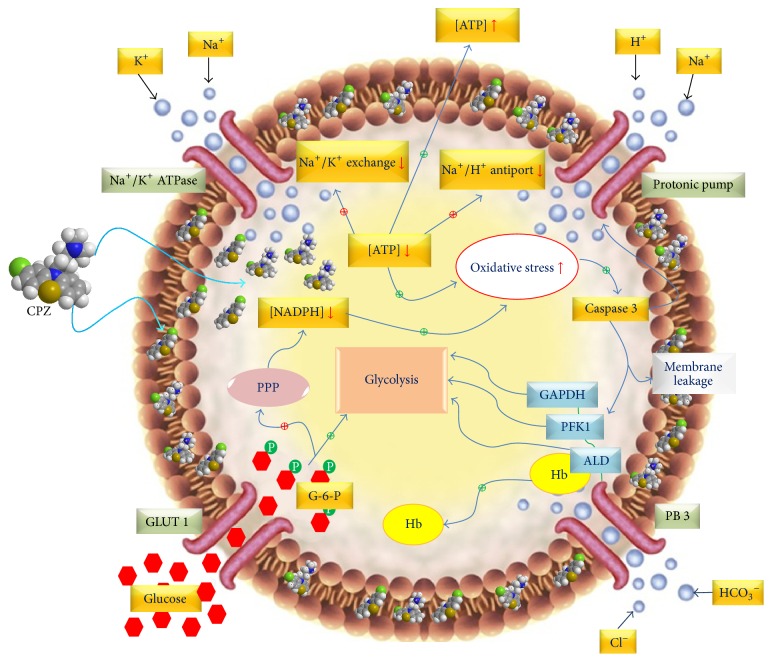
Schematic representation of CPZ effects on RBCs. This scheme has been built taking into account the observation raised by our experimental data and the one obtained from the literature [11, 14, 15, 18–21].

Concluding, the influence of CPZ on RBCs is not limited to the membrane or RBC shape because the drug exerts a number of metabolic effects (Figure 10), which can be useful to counteract specific biological conditions. Caspase 3 activation closely related to oxidative stress and pHi, for instance, as primed stage for apoptotic process could be a promising starting point for the use of CPZ in antiproliferative investigations.

## Figures and Tables

**Table 1:** Effect of CPZ concentrations on the ATP levels in RBCs.

Conditions	*n*	Intracellular [ATP] *μ*M/RBC	Extracellular [ATP] *μ*M/RBC
Control	5	300 ± 0.51	2.7 ± 0.07
CPZ	6	207 ± 0.47	3.9 ± 0.13
Mas	5	318 ± 0.48	3.1 ± 0.08
CPZ + Mas	6	280 ± 0.52	3.6 ± 0.11

**Table 2:** Percentage of met-Hb calculated at different incubation times of RBCs in the absence (control) and in the presence of CPZ 25 μM (a), 50 μM (b), and 100 μM (c). At each incubation time CPZ samples showed significant statistical differences versus control (*P* < 0.05).

Conditions	Incubation time (min)	met-Hb (%) (a)	met-Hb (%) (b)	met-Hb (%) (c)
Control	15	0	0	0
CPZ	15	23 ± 2	25 ± 2	25 ± 2
Control	90	0	0	0
CPZ	90	51 ± 3	60 ± 3	60 ± 3

## Abbreviations

CPZ: Chlorpromazine
PS: Phosphatidylserine
AchE: Acetylcholinesterase
RBCs: Red blood cells
PB3: Band 3 protein
Hb: Hemoglobin
GEs: Glycolytic enzymes
G6P: Glucose-6-phosphate
PPP: Pentose phosphate pathway
HOS: High-oxygenation state
LOS: Low-oxygenation state
DNPH: 2,4-Dinitrophenylhydrazine
p-NPP: p-Nitrophenylphosphate
Met-Hb: Methemoglobin
PMSF: Phenylmethanesulfonylfluoride
NBT: Nitroblue tetrazolium
PMS: Phenazine methosulphate
t-BHT: * tert*-Butyl-hydroperoxide
EDTA: Ethylenediaminetetraacetic acid
TBARS: Thiobarbituric acid reactive substances
OV: Orthovanadate
DNP: Dinitrophenyl
FACS: Fluorescence-activated cell sorting
SD: Standard deviation
pHi: Cytoplasmic pH.
